# Associations between sarcopenia and domains of quality of life in older adults: a population-based cross-sectional study

**DOI:** 10.1186/s12955-025-02358-y

**Published:** 2025-04-14

**Authors:** Emma C. West, Lana J. Williams, Jessica A. Davis, Julie A. Pasco

**Affiliations:** 1 IMPACT– Institute for Mental and Physical Health and Clinical Translation, School of Medicine, Deakin University, Geelong, VIC Australia; 2 Barwon Health, Geelong, VIC Australia; 3 Department of Medicine-Western Health, The University of Melbourne, St Albans, VIC Australia; 4 Department of Epidemiology and Preventative Medicine, Monash University, Melbourne, VIC Australia

**Keywords:** Sarcopenia, Skeletal muscle, Ageing, Quality of life

## Abstract

**Background:**

Sarcopenia is an age-related skeletal muscle disorder associated with deleterious health outcomes. Few studies have examined associations between sarcopenia and quality of life (QoL). Therefore, the purpose of this study was to determine whether sarcopenia is independently associated with specific domains of QoL.

**Methods:**

This cross-sectional study examined associations between sarcopenia and domains of QoL in a population-based sample of 682 adults aged 60–96 years. Sarcopenia was defined according to the revised European Working Group on Sarcopenia in Older People algorithm. Appendicular lean mass was assessed using dual-energy Xray absorptiometry, handgrip strength by dynamometry, and physical performance using the Timed UupandGo test. The World Health Organisation’s abbreviated QoL tool was used to assess QoL across four domains: physical health, psychological, social relationships and environment. Multivariable logistic regression was used to investigate associations between sarcopenia and poor QoL.

**Results:**

After adjusting for potential covariates, sarcopenia (either probable or confirmed) was associated with an increased likelihood for poor physical health-related QoL [OR 2.77 (95% CI 1.31–5.88)] and poor psychological-related QoL [OR 2.69 (95% CI 1.41–5.15)]. No associations were detected between sarcopenia and the social relationships or environment domains.

**Conclusions:**

These findings highlight the importance of maintaining skeletal muscle health in older age. Interventions to prevent or manage sarcopenia among older adults may contribute to better QoL for this population and warrant further investigation.

**Supplementary Information:**

The online version contains supplementary material available at 10.1186/s12955-025-02358-y.

## Introduction

Sarcopenia is an age-related skeletal muscle disorder characterised by an accelerated loss of skeletal muscle mass and function (muscle strength or physical performance) [1]. Prevalence ranges from 10 to 27% in adults aged ≥ 60 years [2] however these figures are estimated to increase dramatically due to current trends in population ageing [3]. A growing body of literature has found sarcopenia to be associated with deleterious health outcomes including increased risk of disability, falls, post-operative complications, and mortality [4]. Consequently, sarcopenia is also associated with decreased health-related quality of life [5].

Quality of life (QoL) is a subjective measure of health that has been defined by the World Health Organisation as “individuals’ perception of their position in life in the context of the culture and value systems in which they live, and in relation to their goals, expectations, standards and concerns” [6]. While objective measures of health focus on disease status, subjective measures of health such as QoL are inherently person-centred. Increasingly, subjective measures of health are being utilised in clinical practice and are considered essential to the care of older adults who commonly have multiple chronic conditions and complex needs [7].

Although QoL is a multidimensional concept, only a few studies have examined the relationship between sarcopenia and specific domains of QoL. Haider et al. [8] examined associations between components of sarcopenia and QoL in a sample of 83 pre-frail and frail community-dwelling older adults using the World Health Organisation’s abbreviated QoL tool (WHOQoL-BREF). Findings indicated that muscle function, as assessed by handgrip strength (HGS), was associated with overall QoL scores. However, no associations were observed between muscle measures and the specific domains of QoL [8]. In a larger sample of older adults receiving nursing care (*n* = 498) Doležalová et al. [9] found that those with sarcopenia had poorer QoL scores across all four WHOQoL-BREF domains (physical health, psychological, social relationships, and environment) compared to those without sarcopenia. This study also reported associations between sarcopenia and frailty, nutritional state, and cognitive status. However, whether the relationship between sarcopenia and QoL is independent of these factors remains unclear as only crude analyses were reported [9].

Studies involving other popular multidimensional QoL assessments in community-dwelling samples have produced mixed results. A large study involving 2,987 community-dwelling men and women from the Hertfordshire Cohort Study found those with lower HGS were more likely to report poor QoL across all eight domains of QoL, as assessed by the 36-item Short Form survey (SF-36). After adjusting for age, height, weight, walking speed, social class, cigarette and alcohol consumption and comorbidities, lower HGS was associated with poor QoL across six domains of QoL (physical functioning, role physical, mental health, vitality, bodily pain, and general health) [10]. Conversely, the SarcoPhAge study assessed QoL among 534 community dwelling adults aged ≥ 65 years and found sarcopenia was only associated with decreased QoL in the physical functioning domain of the SF-36 [11]. Indeed, it has been suggested that among sarcopenic community-dwelling older adults QoL is mainly impacted on physical and functional domains [12].

With a rapidly ageing population and growing focus on subjective measures of health, the effect of age-related diseases on QoL warrants further investigation. Given sarcopenia is an age-related disease that significantly impacts mobility, it is likely to have impacts on QoL beyond physical functioning. People with sarcopenia may experience physical limitations that negatively affect their body image, ability to participate in social activities, or finances via increased healthcare needs. Evaluating specific domains of QoL can provide a more comprehensive understanding of QoL in sarcopenic populations. However, to date, few studies have explored the relationship between sarcopenia and specific domains of QoL. Further, these investigations are largely limited by methodological weaknesses and have produced mixed findings that may not be generalisable to broader populations. Therefore, in the present study, we aimed to determine whether sarcopenia is independently associated with domains of QoL in a population-based sample of men and women. We hypothesised that those with sarcopenia would be more likely to have poor QoL across all domains of the WHOQoL-BREF.

## Materials and methods

### Study design and participants

This cross-sectional study utilised data from women and men enrolled in the Geelong Osteoporosis Study (GOS), a population-based prospective cohort study situated in south-eastern Australia. A detailed description of the GOS cohort and study design has been published previously [13]. Briefly, 1494 women and 1540 men were recruited at random from the Commonwealth electoral rolls for the Barwon Statistical Division, an area comprising regional and urban communities. The inclusion criterion was a listing as a resident on the electoral rolls for the region. As voting is compulsory in Australia, the electoral rolls provide a comprehensive sampling frame of adults aged ≥ 18 years. Exclusion criteria included residency in the region for less than 6 months and inability to provide informed consent. Since its inception in 1993, participants have been invited to undertake serial physical and mental health assessments.

Data for the current study were drawn from the most recently completed assessment phases, with data collected from 2011 to 2014 for women and from 2016 to 2020 for men. From a potential 849 women and 625 men who participated in these phases, 375 women and 367 men were aged ≥ 60 years. Participants were not included in the present study if they did not complete physical or QoL assessments. The final sample included 339 women and 343 men aged between 60 and 96 years.

### Sarcopenia definition

Sarcopenia was defined according to the revised guidelines published by the European Working Group on Sarcopenia in Older People (EWGSOP2) [1]. According to EWGSOP2, probable sarcopenia is determined by low muscle strength and confirmed sarcopenia is determined by the presence of both low muscle strength and low muscle mass. When low muscle strength and low muscle mass are accompanied by poor physical performance, sarcopenia is considered severe.

Handgrip strength (HGS) (kg) was measured using a hand-held dynamometer (Jamar, Sammons Preston, Bolingbrook, IL, USA, for women; Vernier, LoggerPro3, USA for men) with the participant seated and the arm flexed at the elbow at 90-degree angle. Maximum HGS was taken for each hand in duplicate for women and triplicate for men. Measures from the Vernier device were transformed to Jamar equivalent values according to the following equation: HGSJamar (kg) = 9.50 + 0.818 * HGSVernier (kg) + 8.80 * Sex, where sex = 1 for men. This equation was developed by measuring the maximum HGS on each device for 45 men and women aged 21–67 years, as previously described [14]. Lean mass, a proxy measure for muscle mass, was measured using dual-energy X-ray absorptiometry (DXA; Lunar Prodigy Pro, Madison, WI, USA), which provided measures for appendicular lean mass (ALM). Appendicular lean mass index (ALMI) was calculated as the ALM/height squared (kg/m^2^). Height was measured to the nearest 0.1 cm using a Harpenden stadiometer and body weight to the nearest 0.1 kg. Both measurements were taken with participants wearing minimal clothing and no shoes. Body mass index (BMI) was calculated as weight/height squared (kg/m^2^). Physical performance was assessed using the Timed Up and Go (TUG) test over a distance of three meters [15].

As per the EWGSOP2 definition [1], low HGS was considered as < 27 kg for men and < 16 kg for women; low ALMI was considered < 7.0 kg/m^2^ for men and < 5.5 kg/m^2^ for women; and slow TUG ≥ 20 s for both men and women.

### Quality of life

The Australian version of the WHOQoL-BREF was used to assess QoL [16]. The WHOQoL-BREF is a 26-item tool which contains four domains of QoL: physical health, psychological, social relationships, and environment. The physical health domain includes seven items pertaining to activities of daily living, dependence on medication, energy, mobility, pain, sleep, and work capacity. The psychological domain includes six items related to bodyimage, negative feelings, positive feelings, self-esteem, spirituality or religion, and cognition. The social relationships domain contains three items related to personal relationships, social support, and sex life. The environment domain contains eight items related to financial resources, safety, health and social services, home environment, opportunities to acquire new skills and knowledge, recreation, transport, and physical environment (i.e., pollution, noise, traffic, and climate). It also contains two global items pertaining to overall QoL and general health. All items are rated on a 5-point Likert scale with scores scaled in a positive direction (i.e., higher scores indicate a higher QoL). The WHOQoL-BREF has demonstrated high reliability and validity for several populations worldwide [17].

### Covariates

Potentially confounding sociodemographic and lifestyle variables included age, socio-economic status (SES), marital status, smoking status, diet quality, alcohol use, and physical activity level. The Australia Bureau of Statistics Index of Relative Socio-economic Advantage and Disadvantage (IRSAD) was used to determine area-based SES [18]. IRSAD quintiles 1 and 2 were pooled to create the low SES group and quintiles 4 and 5 were pooled to create the upper SES group. Quintile 3 was categorised as mid SES. Marital status and smoking status were self-reported. Marital status was categorised as currently “married/in a relationship” or “other.” Smoking status was categorised as “ever smoked” or “never smoked”. The Australian Cancer Council’s Dietary Questionnaire for Epidemiological Studies (DQES) was employed to collect dietary data [19]. The DQES collects information on the quantity and frequency of food and alcohol consumption in the preceding 12 months. From the DQES data, the Australian Recommended Food Score (ARFS) was calculated as an indicator of diet quality. The ARFS focuses on dietary variety within food groups recommended in the Australian Dietary Guidelines with total scores ranging from zero to 73 [20]. Alcohol consumption was also derived from DQES data and average consumption categorised as > 14.3 g/day. A cut-off of > 14.3 g indicates alcohol consumption that exceeds recommendations from Australian Alcohol Guidelines. Habitual physical activity was measured using the Elderly Physical Activity Questionnaire. The questionnaire included items on household activities, sports, and leisure time over a 12-month period [21]. Symptoms of depression and anxiety were measured using the Hospital Anxiety and Depression Scale (HADS). The HADS contains 14 items and has two subscales: anxiety and depression. Total HADS scores for each subscale were dichotomised, whereby a score of ≥ 8 on the depression subscale (HADS-D) indicated the presence of depression and a score of ≥ 8 on the anxiety subscale (HADS-A) indicated the presence of anxiety [22].

### Statistical analysis

All statistical analyses were performed using Stata v.17 (College Station, TX: StataCorp). Descriptive statistics were used to describe participant characteristics for the whole sample and according to sarcopenia status. Differences between the groups were identified using analysis of variance (ANOVA) where continuous data were normally distributed and the Kruskall-Wallis test where continuous data deviated from the normal distribution. For categorical data, the Chi-square test was used, employing Fisher’s exact test where expected cell counts were < 5.

Logistic regression (odds ratios with 95% confidence intervals) was undertaken to investigate the association between sarcopenia and domains of the WHOQoL-BREF. Scores on each domain of the WHOQoL-BREF were dichotomised as low and high according to published norms for each sex. For women: physical health *M* = 72.94, psychological *M* = 68.88, social relationships *M* = 72.31, environment *M* = 79.16. For men: physical health *M* = 75.93, psychological *M* = 74.92, social relationships *M* = 73.37, environment *M* = 80.17 [23]. Best models were identified by backward elimination after considering potential covariates; variables were retained in the model if *p* < 0.05. Final models were tested for effect modification.

## Results

Based on the EWGSOP2 algorithm, 45 participants (6.6%) met criteria for probable sarcopenia (low HGS) and 12 (1.8%) participants met criteria for confirmed sarcopenia (both low ALM and low HGS). Of those with confirmed sarcopenia, only two participants were considered to have severe sarcopenia with a TUG score of ≥ 20 s. Descriptive characteristics for the entire sample according to sarcopenia status are presented in Table 1.

**Table 1:** Participant characteristics for the whole group and according to sarcopenia status

Characteristic	All (*n* = 682)	No sarcopenia (*n* = 625)	Probable sarcopenia (*n* = 45)	Sarcopenia (*n* = 12)	*p*		
Sex (female) (n, %)	339 (49.7)	294 (47.0)	37 (82.2)	8 (66.7)	< 0.001		
Age (year) (median, IQR)	70.9 (65.5–78.1)	70.4 (65.1–76.9)	82.2 (74.0-87.5)	74.3 (67.3–82.2)			
SES (n, %)					0.250		
Low	174 (25.7)	165 (25.5)	7 (15.9)	2 (16.7)			
Mid	339 (50.0)	304 (48.9)	26 (59.1)	9 (75.0)			
Upper	165 (24.3)	153 (24.6)	11 (25.0)	1 (8.3)			
Married/in relationship (n, %)	489 (71.9)	465 (74.4)	17 (39.5)	7 (58.3)	< 0.001		
Education (n, %)					0.017		
No high school	328 (48.3)	288 (46.2)	33 (75.0)	7 (58.3)			
Completed high school	81 (11.9)	76 (12.2)	3 (6.8)	2 (16.7)			
TAFE	172 (25.3)	164 (26.3)	6 (13.6)	2 (16.7)			
University	98 (14.4)	95 (15.3)	2 (4.6)	1 (8.3)			
BMI (kg/m^2^) (mean, SD)	28.4 (5.4)	28.4 (5.3)	29.2 (6.8)	23.5 (3.1)	0.004		
Smoker (ever) (n, %)	292 (42.9)	269 (43.1)	18 (40.0)	5 (41.7)	0.917		
Physical activity (median, IQR)	7.2 (2.4–16.2)	8.4 (2.8–16.9)	1.9 (1.2–5.3)	2.3 (1.3–13.9)	< 0.001		
Alcohol ≥ 20 g/day (n, %)	259 (38.7)	249 (40.6)	7 (16.3)	3 (25.0)	0.004		
Diet (ARFS) (mean, SD)	32.1 (9.3)	32.2 (9.3)	31.8 (8.4)	27.6 (11.0)	0.276		
HADS-D ≥ 8 (n, %)	56 (8.2)	44 (7.0)	9 (20.0)	3 (25.0)	0.002		
HADS-A ≥ 8 (n, %)	122 (17.9)	110 (17.6)	7 (15.6)	5 (41.7)	0.090		
Poor QoL (WHOQOL-BREF) (n, %)							
Physical health	379 (55.7)	335 (53.6)	34 (77.3)	10 (83.3)	0.001		
Psychological	267 (39.2)	233 (37.3)	28 (63.6)	6 (50.0)	0.002		
Social relationships	286 (42.8)	251 (41.0)	27 (61.4)	8 (66.7)	0.007		
Environment	305 (45.0)	271 (43.6)	29 (65.9)	5 (41.7)	0.015		

ARFS, Australian Recommended Food Score; BMI, Body Mass Index; HADS, Hospital Anxiety and Depression Scale; SES, Socioeconomic status; QoL, quality of life; WHOQoL-BREF, World Health Organisation Quality of Life Brief assessment tool

Missing: SES = 4, relationship status = 2, education = 3, BMI = 8, physical activity = 3, diet = 13, QOL: physical health = 1, psychological = 1, social relationships = 13, environment = 4

The unadjusted odds ratios (ORs) for QoL domains according to sarcopenia status are presented in Table 2. Probable sarcopenia (low HGS) was associated with an increased likelihood for poor QoL across all four WHOQoL-BREF domains. No association between confirmed sarcopenia and QoL domains was observed. Given the small proportion of participants with sarcopenia, participants with probable, confirmed, and severe sarcopenia were pooled in multivariable logistic regression analysis. After adjusting for education, marital status, physical activity, smoking status, BMI, and symptoms of depression and anxiety, sarcopenia was associated with an increased likelihood for poor physical health-related QoL [OR 2.77 (95% CI 1.31–5.88), *p* = 0.008]. Sarcopenia was also associated with an increased likelihood of poor psychological-related QoL [OR 2.69 (95% CI 1.41–5.15), *p* = 0.003] adjusting for SES, smoking status, and symptoms of depression and anxiety. No associations between sarcopenia status and the social relationships or environment domains of QoL were observed.

**Table 2:** Unadjusted associations between sarcopenia and WHOQoL-BREF domains

	Physical health		Psychological		Social Relationships		Environment	
WHOQoL-BREF domain	Odds ratio (95% CI)	*p*	Odds ratio (95% CI)	*p*	Odds ratio (95% CI)	*p*	Odds ratio (95% CI)	*p*
No sarcopenia	Reference	-	Reference	-	Reference	-	Reference	-
Probable sarcopenia	2.94 (1.43–6.06)	0.003	2.94 (1.56–5.56)	0.001	2.29 (1.22–4.29)	0.010	2.50 (1.32–4.76)	0.005
Confirmed sarcopenia	4.33 (0.94–19.92)	0.060	1.68 (0.54–5.28)	0.372	2.88 (0.86–9.68)	0.086	0.93 (0.29–2.95)	0.895

## Discussion

### Sarcopenia and quality of life

In this population-based sample of men and women, we examined the relationship between sarcopenia and domains of QoL, as assessed by the WHOQoL-BREF. We found sarcopenia was associated with an increased likelihood for poor physical health- and psychological-related QoL, independent of sociodemographic and lifestyle risk factors. To the best of our knowledge, this is the first study to examine associations between sarcopenia and WHOQoL-BREF domains in a representative sample. Our findings are consistent with other studies that have identified associations between sarcopenia and domains of QoL beyond physical functioning. Doležalová and colleagues found sarcopenia was associated with lower QoL across all four WHOQoL-BREF domains, with largest effect sizes observed in the physical and psychological domains of QoL [9]. Similarly, Sayer et al. found decreases in HGS were associated with poorer QoL in both the physical functioning and mental health domains of the SF-36 [10].

In the current study, we report that older adults with sarcopenia were almost three times more likely to experience poor QoL in the physical health domain. Given skeletal muscle is necessary for locomotion and performance of daily activities of living this finding is not unexpected. The progressive loss of muscle mass, muscle function, and muscle quality that results from sarcopenia may emerge as mobility impairments, disability, bodily pain, and loss of independence. Indeed, data from the Rural Frailty Study in Mexico suggests poor QoL is most evident among those with severe sarcopenia [24]. However, within the current study, only two participants satisfied criteria for severe sarcopenia. This would suggest that sarcopenia, even in its early stages may have a deleterious impact on physical health-related QoL.

Perhaps most interesting is the finding that older adults with sarcopenia were 2.7-fold more likely to have poor psychological-related QoL. Previous work suggests sarcopenia may be associated with indicators of psychological health and wellbeing beyond QoL. For example, data from the Korea National Health and Nutrition Examination Survey found sarcopenic obesity, a condition where sarcopenia is concomitant with obesity, was associated with higher levels of perceived stress and suicidal ideation [25]. Another study utilising data from the GOS reported low muscle strength was associated with an increased likelihood of low cognition among postmenopausal women [26].

Due to the features captured in the psychological QoL domain (e.g., mood) it is plausible that lower psychological-related QoL is reflective of mental health conditions. The extant literature demonstrates an association between poor muscle function and depression [27]. As such, it has been suggested that operational definitions of sarcopenia should consider depressive symptoms at the time of assessment [28]. However, in the current study, we found symptoms of depression and anxiety did not attenuate the relationship between sarcopenia and poor psychological QoL. Thus, it appears sarcopenia is associated with poor psychological QoL independent of mental illness. This aligns with previous work which suggests sarcopenia is independently associated with both QoL and depressive symptoms [29].

Poor QoL has numerous clinical implications and is increasingly recognised as crucial to patient outcomes. Among patients with cancer, QoL at baseline is frequently found to be significant prognostic factor of survival [30]. QoL has also been associated with other factors that may lead to the worsening of physical health, such as low adherence to prescribed treatment [31]. Addressing poor QoL is of critical importance and investigating the specific domains of QoL rather than overall scores allows for a more nuanced understanding of QoL upon which interventions can be tailored and enhanced. Interventions designed to build muscle strength may have the double benefit of preventing sarcopenia and improving QoL in older adults. Resistance exercise training (RET) is currently considered a first-line treatment for managing and preventing sarcopenia [32] and has similarly demonstrated effectiveness in increasing QoL among older adults [33]. Given our finding that sarcopenia is independently associated with poor psychological related QoL, psycho-social interventions may be a useful addition to mainstay interventions such as RET. Cognitive behavioural therapy is one psychological intervention with demonstrated effectiveness in addressing concerns associated with other musculoskeletal conditions such as knee/hip osteoarthritis [34]. However, further research is needed to understand whether psycho-social interventions are of value to people with sarcopenia.

The impact of sarcopenia not only on QoL but on other measures of psychological well-being warrants further investigation. Future studies examining the impact of RET to improve or prevent sarcopenia should consider the inclusion of QoL and psychological wellbeing as outcomes of interest. It has been demonstrated that traditional psychometric criteria likely conflict with the complexity of clinical reality. Clinimetric Patient-Reported Outcome Measures (CLIPROM) criteria have been proposed to address this issue. Measures with highly valid clinimetric indices, such as the WHO-5 and the SCL-90-R may be particularly useful tools to employ in future research [35, 36].

### Strengths and limitations

The use of a randomly selected population-based sample rather than selection based on disease is a clear strength of this study. Loss to follow-up and inability to participate in a clinical exam are limitations that may have contributed to the presence of a healthy participant bias and underestimation of results. Due to the small proportion of participants with sarcopenia we were unable to compare sarcopenia at different stages. Future studies should aim to examine the relationship between sarcopenia and domains of QoL in larger samples.

Sarcopenia status was determined using gold standard methods and QoL was assessed using an internationally validated tool covering diverse aspects of life circumstances. Unlike other QoL tools, the WHOQoL-BREF allows for the evaluation of specific domains of QoL. However, we acknowledge that QoL domains do not exist independently and are interconnected. For example, social relationships are known to play an important role in psychological wellbeing [37]. Furthermore, given the highly subjective nature of QoL, evaluation across the literature is heterogenous making direct comparisons between studies difficult.

The cross-sectional design of the study meant it was not possible to determine a causal relationship between sarcopenia and QoL. It is plausible that poor QoL leads to unfavourable lifestyle choices such as physical inactivity and poor diet which could in turn trigger a decline in muscle mass and quality [38]. We acknowledge that the self-report measures may have led to information bias. Measures of diet and alcohol consumption were based on a 12-month food frequency questionnaire whereas a more granular dietary assessment may be more informative, given many older adults will make lifestyle changes after receiving a diagnosis or experiencing declining health.

## Conclusion

In this population-based sample of men and women aged ≥ 60 years, sarcopenia was associated with an increased likelihood for poor physical- and psychological-related QoL. Overall, our findings reinforce the important role skeletal muscle mass and function has on QoL. Interventions to prevent or manage loss of muscle function among older adults, such as resistance exercise training, may contribute to better QoL for this population.

## Electronic supplementary material

Below is the link to the electronic supplementary material.

Supplementary Material 1

Supplementary Material 2

## Data Availability

No datasets were generated or analysed during the current study.

## Abbreviations

ALM: Appendicular lean mass
ALMI: Appendicular lean mass index
ARFS: Australian recommended food score
DQES: Dietary questionnaire for epidemiological studies
EWGSOP2: European working group on sarcopenia in older people, revised definition
GOS: Geelong osteoporosis study
HADS: Hospital anxiety and depression scale
HGS: Handgrip strength
IRSAD: Index of relative socio-economic advantage and disadvantage
OR: Odds ratio
QoL: Quality of life
RET: Resistance exercise training
SES: Socio-economic status
TUG: Timed Up and Go test
WHOQoL-BREF: World health organisation’s abbreviated QoL tool
