# Supplementary material for: Associations between sarcopenia and domains of quality of life in older adults: a population-based cross-sectional study
Source: Health Qual Life Outcomes. 2025 Apr 14;23:38. doi: 10.1186/s12955-025-02358-y (PMC11998198; doi:10.1186/s12955-025-02358-y)
Supplement: Supplementary file 1 — Supplementary Material 1 [file 12955_2025_2358_MOESM1_ESM.docx]

**Table 1. Participant characteristics for the whole group and according to sarcopenia status**

| Characteristic | All  (n = 682) | No sarcopenia  (n = 625) | Probable sarcopenia  (n = 45) | Sarcopenia  (n =12) | p |  |  |
| --- | --- | --- | --- | --- | --- | --- | --- |
| Sex (female) (n, %) | 339 (49.7) | 294 (47.0) | 37 (82.2) | 8 (66.7) | <0.001 |  |  |
| Age (year) (median, IQR) | 70.9 (65.5-78.1) | 70.4 (65.1-76.9) | 82.2 (74.0-87.5) | 74.3 (67.3-82.2) |  |  |  |
| SES (n, %) | | | | | 0.250 | | |
| Low | 174 (25.7) | 165 (25.5) | 7 (15.9) | 2 (16.7) |  |  |  |
| Mid | 339 (50.0) | 304 (48.9) | 26 (59.1) | 9 (75.0) |  |  |  |
| Upper | 165 (24.3) | 153 (24.6) | 11 (25.0) | 1 (8.3) |  |  |  |
| Married/in relationship (n, %) | 489 (71.9) | 465 (74.4) | 17 (39.5) | 7 (58.3) | <0.001 |  |  |
| Education (n, %) |  |  |  |  | 0.017 |  |  |
| No high school | 328 (48.3) | 288 (46.2) | 33 (75.0) | 7 (58.3) |  |  |  |
| Completed high school | 81 (11.9) | 76 (12.2) | 3 (6.8) | 2 (16.7) |  |  |  |
| TAFE | 172 (25.3) | 164 (26.3) | 6 (13.6) | 2 (16.7) |  |  |  |
| University | 98 (14.4) | 95 (15.3) | 2 (4.6) | 1 (8.3) |  |  |  |
| BMI (kg/m^2^) (mean, SD) | 28.4 (5.4) | 28.4 (5.3) | 29.2 (6.8) | 23.5 (3.1) | 0.004 |  |  |
| Smoker (ever) (n, %) | 292 (42.9) | 269 (43.1) | 18 (40.0) | 5 (41.7) | 0.917 |  |  |
| Physical activity (median, IQR) | 7.2 (2.4-16.2) | 8.4 (2.8-16.9) | 1.9 (1.2-5.3) | 2.3 (1.3-13.9) | <0.001 |  |  |
| Alcohol ≥ 20g/day (n, %) | 259 (38.7) | 249 (40.6) | 7 (16.3) | 3 (25.0) | 0.004 |  |  |
| Diet (ARFS) (mean, SD) | 32.1 (9.3) | 32.2 (9.3) | 31.8 (8.4) | 27.6 (11.0) | 0.276 |  |  |
| HADS-D ≥8 (n, %) | 56 (8.2) | 44 (7.0) | 9 (20.0) | 3 (25.0) | 0.002 |  |  |
| HADS-A ≥8 (n, %) | 122 (17.9) | 110 (17.6) | 7 (15.6) | 5 (41.7) | 0.090 |  |  |
| Poor QoL (WHOQOL-BREF) (n, %) | | | | | | |  |
| Physical health | 379 (55.7) | 335 (53.6) | 34 (77.3) | 10 (83.3) | 0.001 |  |  |
| Psychological | 267 (39.2) | 233 (37.3) | 28 (63.6) | 6 (50.0) | 0.002 |  |  |
| Social relationships | 286 (42.8) | 251 (41.0) | 27 (61.4) | 8 (66.7) | 0.007 |  |  |
| Environment | 305 (45.0) | 271 (43.6) | 29 (65.9) | 5 (41.7) | 0.015 |  |  |

ARFS, Australian Recommended Food Score; BMI, Body Mass Index; HADS, Hospital Anxiety and Depression Scale; SES, Socioeconomic status; QoL, quality of life; WHOQoL-BREF, World Health Organisation Quality of Life Brief assessment tool.

Missing: SES=4, relationship status=2, education=3, BMI=8, physical activity=3, diet=13, QOL: physical health=1, psychological=1, social relationships=13, environment=4

**Table 2. Unadjusted associations between sarcopenia and WHOQoL-BREF domains**

|  | Physical health |  | Psychological |  | Social Relationships |  | Environment |  |
| --- | --- | --- | --- | --- | --- | --- | --- | --- |
| WHOQoL-BREF domain | Odds ratio (95% CI) | *p* | Odds ratio (95% CI) | *p* | Odds ratio (95% CI) | *p* | Odds ratio (95% CI) | *p* |
| No sarcopenia | Reference | - | Reference | - | Reference | - | Reference | - |
| Probable sarcopenia | 2.94 (1.43-6.06) | 0.003 | 2.94 (1.56-5.56) | 0.001 | 2.29 (1.22-4.29) | 0.010 | 2.50 (1.32-4.76) | 0.005 |
| Confirmed sarcopenia | 4.33 (0.94-19.92) | 0.060 | 1.68 (0.54-5.28) | 0.372 | 2.88 (0.86-9.68) | 0.086 | 0.93 (0.29-2.95) | 0.895 |

**Table 3. Adjusted associations between sarcopenia (probable or confirmed) and WHOQoL-BREF domains**

|  | Physical health |  | Psychological |  | Social Relationships |  | Environment |  |
| --- | --- | --- | --- | --- | --- | --- | --- | --- |
| WHOQoL-BREF domain | Odds ratio (95% CI) | *p* | Odds ratio (95% CI) | *p* | Odds ratio (95% CI) | *p* | Odds ratio (95% CI) | *p* |
| No sarcopenia | Reference | - | Reference | - | Reference | - | Reference | - |
| Confirmed sarcopenia | 4.33 (0.94-19.92) | 0.060 | 1.68 (0.54-5.28) | 0.372 | 2.88 (0.86-9.68) | 0.086 | 0.93 (0.29-2.95) | 0.895 |
